# Supplementary material for: MetaRibo-Seq measures translation in microbiomes
Source: Nat Commun. 2020 Jun 29;11:3268. doi: 10.1038/s41467-020-17081-z (PMC7324362; doi:10.1038/s41467-020-17081-z)
Supplement: Supplementary file 10 — Supplementary Data 7 [file 41467_2020_17081_MOESM10_ESM.zip › File2/Confidence_High_Taxonomy/241046_out.krona.html]

Javascript must be enabled to view this page.

members
magnitude
magnitudeUnassigned
count
unassigned
taxon
rank

241046\_out

1

SRS050752\_contig\_number\_21385
53


SRS014412\_contig\_number\_14794
36
1
2
superkingdom

2
phylum
976

2
class
117743

200644
2
order

2
family
49546

genus
2
76831

SRS143372\_contig\_number\_contig-100\_211.93202SRS149075\_contig\_number\_contig-100\_1079.120308

1224
3
phylum

class
2
28216

206351
2
order

1499392
2
family

535
2
genus


SRS064276\_contig\_number\_17829SRS893369\_contig\_number\_9216
251747
2
species

1
class
1236

1
order
91347

543
1
family

561
1
genus


SRS1055022\_contig\_number\_4407
562
1
species

1239
30
phylum

class
30
186801

30

SRS019496\_contig\_number\_14894SRS076756\_contig\_number\_11974SRS104693\_contig\_number\_11783
order
186802
3

31979
family
2

1485
genus
2


SRS075078\_contig\_number\_contig-100\_1036.188389
59620
1
species

1
species

SRS056273\_contig\_number\_24293
2293011

186803
family
25

841
genus
1

2049040

SRS049773\_contig\_number\_32608
species
1

species
21
1898203

SRS011134\_contig\_number\_36981SRS013951\_contig\_number\_1522SRS015579\_contig\_number\_21322SRS017433\_contig\_number\_17268SRS017916\_contig\_number\_11214SRS019161\_contig\_number\_35835SRS024625\_contig\_number\_19248SRS047433\_contig\_number\_12584SRS075984\_contig\_number\_contig-100\_71.161667SRS077641\_contig\_number\_16683SRS077730\_contig\_number\_15800SRS078242\_contig\_number\_31199SRS105153\_contig\_number\_20559SRS142542\_contig\_number\_13744SRS142890\_contig\_number\_44139SRS142980\_contig\_number\_6335SRS143342\_contig\_number\_3549SRS147977\_contig\_number\_3580SRS148817\_contig\_number\_4144SRS148874\_contig\_number\_contig-100\_226.107950SRS971276\_contig\_number\_12059

572511
2
genus


SRS147425\_contig\_number\_3505
1262756
1
species

species
1
40520

SRS022609\_contig\_number\_contig-100\_2032.233903

189330
1
genus

species
1
1235798

SRS016954\_contig\_number\_contig-100\_5483.5483

2
superkingdom
2759

4751
kingdom
2

2
subkingdom
451864

phylum
1
4890

subphylum
1
147538

147545
class
1

1
subclass
451870

1
order
146291

1
family
146292

364710
1
genus

1
species

SRS1041090\_contig\_number\_2953
364733

phylum
1
5204

29000
1
subphylum

162481
1
class

231213
order
1

1799696
1
family

genus
1
5001

5005

SRS893231\_contig\_number\_490
species
1

14

SRS014948\_contig\_number\_1380SRS020233\_contig\_number\_34301SRS021948\_contig\_number\_contig-100\_2576.163754SRS024331\_contig\_number\_contig-100\_4217.165842SRS045826\_contig\_number\_contig-100\_19409.19410SRS053356\_contig\_number\_43145SRS077335\_contig\_number\_contig-100\_14644.14644SRS098061\_contig\_number\_contig-100\_2091.40323SRS100021\_contig\_number\_contig-100\_4551.4551SRS1041031\_contig\_number\_23795SRS1041146\_contig\_number\_contig-100\_2105.2106SRS143876\_contig\_number\_39161SRS144714\_contig\_number\_21366SRS893230\_contig\_number\_contig-100\_4404.58994
